# Determination of the Copy Number of Porcine Endogenous Retroviruses (PERV) in Auckland Island Pigs Repeatedly Used for Clinical Xenotransplantation and Elimination of PERV-C

**DOI:** 10.3390/microorganisms12010098

**Published:** 2024-01-03

**Authors:** Uwe Fiebig, Luise Krüger, Joachim Denner

**Affiliations:** 1 Robert Koch Institute, 13353 Berlin, Germany; fiebigu@rki.de (U.F.); luise.krueger@web.de (L.K.); 2 Institute of Virology, Free University, 14163 Berlin, Germany

**Keywords:** porcine endogenous retroviruses, Auckland Island pigs, islet cell xenotransplantation

## Abstract

Auckland Island pigs represent an inbred population of feral pigs isolated on the sub-Antarctic island for over 100 years. The animals have been maintained under pathogen-free conditions in New Zealand; they are well characterized virologically and have been used as donor sources in first clinical trials of porcine neonatal islet cell transplantation for the treatment of human diabetes patients. The animals do not carry any of the xenotransplantation-relevant viruses, and in the first clinical trials, no porcine viruses, including porcine endogenous retroviruses (PERVs) were transmitted to the human recipients. PERVs pose a special risk in xenotransplantation, since they are part of the pig genome. When the copy number of PERVs in these animals was analyzed using droplet digital PCR and primers binding to a conserved region of the polymerase gene (PERVpol), a copy number typical for Western pigs was found. This confirms previous phylogenetic analyses of microsatellites as well as mitochondrial analyses showing a closer relationship to European pigs than to Chinese pigs. When kidney cells from very young piglets were analyzed, only around 20 PERVpol copies were detected. Using these cells as donors in somatic cell nuclear transfer (SCNT), animals were born showing PERVpol copy numbers between 35 and 56. These data indicate that Auckland Island pigs have a similar copy number in comparison with other Western pig breeds and that the copy number is higher in adult animals compared with cells from young piglets. Most importantly, PERV-C-free animals were selected and the absence of an additional eight porcine viruses was demonstrated.

## 1. Introduction

The Auckland Islands are a group of sub-Antarctic islands that lie some 560 km south of New Zealand. Pigs were originally introduced onto these islands in 1807 by Captain Abraham Bristow, and in 1840, James Ross brought more pigs onto the island [1,2]. From the time of the last release, the pig population on Auckland Island remained isolated for the next hundred years. Pigs were removed from Auckland Island in 1999 to help restore the environment to its former state, and were brought to the mainland of New Zealand for conservation. A total of 17 animals were housed, initially in quarantine, in a special, purpose-built facility in Invercargill, where they have been successfully bred [3]. The pigs are black or white to brown with black markings, and have a long snout and high hair and bristle coverage for the cold conditions [1]. When the mitochondrial D-loop DNA sequences of the Auckland Island pigs were compared with sequences from domestic and wild boar, the sequence from the Auckland Island pigs clustered with domestic European breeds, and they represent a single breeding population [1,2]. Phylogenetic analyses of microsatellites also showed that they are more closely related to European pigs than Chinese pigs, which is consistent with the mitochondrial analyses [3].

These pigs have been monitored for 8 years and were found negative for all tested porcine infectious agents [4,5,6,7]. Among the tested viruses potentially posing a risk for xenotransplantation were the porcine endogenous retrovirus (PERV), porcine cytomegalovirus (PCMV), a porcine roseolovirus (PCMV/PRV), porcine lymphotropic herpesvirus (PLHV), hepatitis E virus (HEV), porcine teschovirus (PTV), porcine rotaviruses A, B, C (PRVA, B, C), porcine hemagglutinating encephalomyelitis virus (PHEV), porcine parvovirus (PPV), encephalomyocarditis virus (EMCV), porcine enterovirus B (PEVB), porcine reproductive and respiratory syndrome virus (PRRSV), and porcine circovirus (PCV). For testing, diagnostic PCR and serology methods were applied. In comparison with other pig breeds, the Auckland Island pigs are the “cleanest” pigs concerning virological safety. This was the reason to use pig islets from Auckland Island pigs for the treatment of humans with diabetes. In a prospective preclinical trial, transplanting islet cells from Auckland Island pigs into non-human primates, no transmission of pig viruses was observed [6]. Most importantly, no transmission of pig viruses, including PERVs, was observed in two clinical trials performed in New Zealand [7] and Argentina [8].

Here, we analyze the number of PERV proviruses in the genome of adult Auckland Island pigs, in cells from young piglets as well as in animals obtained by somatic cell nuclear transfer (SCNT) using these piglet cells. Since endogenous retroviruses theoretically should behave like cellular genes, we thought it did not matter which organ we used to determine the copy number. However, this conclusion was wrong. Furthermore, we screened these animals for an additional eight xenotransplantation-relevant viruses, among them PCMV/PRV. PCMV/PRV is of special interest, since it is known to significantly reduce the survival time of non-human primates after transplantation of PCMV/PRV-positive kidneys or hearts [9,10,11]. PCMV/PRV was also transmitted in the first clinical trial transplanting a pig heart into a patient in Baltimore, and contributed to the death of the patient [12,13]. We confirmed the absence of PCMV/PRV and other potentially zoonotic viruses in the Auckland Island pigs.

It is known that PERV-C can recombine with PERV-A in living pigs, and the resulting PERV-A/C recombinant viruses are characterized by higher virus titers compared with the paternal PERV-A [14,15]. The release of human tropic PERV-A/C from mitogen-triggered peripheral blood mononuclear cells (PBMCs) was observed in the case of different minipigs as well as ill pigs [16,17,18,19]. Since PERV-C is in contrast to PERV-A and PERV-B not present in all pigs, we screened the Auckland Island pigs for PERV-C and selected PERV-C animals for further breeding. The absence of PERV-C will prevent a recombination with PERV-A.

## 2. Materials and Methods

### 2.1. Animals and Cells

Auckland Island pigs were bred by the NZ Xeno Ltd., Auckland, New Zealand, as described [4,5]. DNA and blood from two groups of animals were sent to Berlin and the PERV copy numbers were analyzed. Later, primary kidney cells from four animals (two male, two female) at an age of about 4 weeks were sent to Germany, to the Ludwig Maximilian University of Munich, and DNA was sent to us to analyze the PERV copy number. Somatic cell nuclear transfer (SCNT) was performed using in vitro matured oocytes, which were collected from abattoir-derived ovaries from German Landrace/Piétrain hybrid gilts. SCNT was performed at the Chair for Molecular Animal Breeding and Biotechnology, and Center for Innovative Medical Models (CiMM) of the Ludwig Maximilian University of Munich. Prior to SCNT, matured oocytes were washed in 0.25% trypsin medium to avoid transmission of pathogens from abattoir. The embryos were transferred laparoscopically to estrus-synchronized recipient sows. Seven piglets were obtained from 373 reconstructed embryos. All piglets were clinically healthy and developed normally. Blood samples taken from 2-week-old piglets and the foster mothers were sent to Berlin and were examined for the presence of PERV-C. In addition, DNA was obtained from ear punches of the animals of the F0 generation at age 416 days and from kidney cells of the F1 generation at age of 70 days, sent to Berlin, and analyzed for the PERV copy number. As an internal standard, the porcine embryonic kidney cell line PK15 (ACC 640, from the Leibniz Institute DSMZ German Collection of Microorganisms and Cell lines, Braunschweig, Germany) was used.

### 2.2. DNA Isolation

DNA was extracted at XenoNZ, New Zealand; at CiMM, Munich, Germany; and by us, using two DNA extraction methods: DNeasy Blood and Tissue kit (Qiagen GmbH, Hilden, Germany) or phenol chloroform extraction using TRIzol Reagent (Invitrogen, Carlsbad, CA, USA) following the manufacturer′s instructions. DNA was quantified using a Qubit 3.0 Fluorometer (Thermo Fischer, Waltham, MA, USA), and the 260 nm/280 nm ratio was determined using a NanoDrop ND-1000 (Thermo Fisher Scientific Inc., Worcester, MA, USA).

### 2.3. Droplet Digital PCR

Droplet digital PCR (ddPCR) was performed as previously described [20,21]. A QX200 droplet generator and a QX100 droplet reader (Bio-Rad) were used and the manufacturer’s instructions (Bio-Rad, Hercules, CA, USA, [http://www.bio-rad.com/de-de/applications-technologies/droplet-digital-pcr-ddpcr-technology?ID=MDV31M4VY, accessed on 19 December 2023) were applied. Purified genomic DNA (100 ng genomic DNA) was digested with 20U MseI (New England Biolabs, Ipswich, MA, USA) at 37 °C for 1 h. Afterwards, the restriction enzyme was heat-inactivated. To perform the ddPCR, the digested DNA was diluted to 5–10 ng/μL. The ddPCR mix consisted of 10 μL 2X ddPCR Master mix, 1.8 μL of each 10 μmol/L target primers (Table 1), 0.5 μL of each 10 μmol/L fluorescent probes (FAM/HEX) (Table 1). A total of 2.5–10 ng digested DNA and water were added to a total volume of 20 μL. Samples were placed into the QX200 Droplet Generator, which utilizes proprietary reagents and microfluidics to partition the samples into 10,000–20,000 nanoliter-sized droplets. Droplets were transferred to a 96-well plate for PCR amplification in an Eppendorf Mastercycler X50 (Eppendorf, Germany). The following cycling conditions were used: 10 min initial enzyme activation at 95 °C, 40× [30 s denaturation at 94 °C, 30 s annealing] followed by a 10 min final elongation step at 98 °C. Following PCR amplification, the samples were placed in the QX200 Droplet Reader (Bio-Rad, Hercules, CA, USA), which analyzes each droplet individually using a two-color detection system (set to detect FAM and either HEX), enabling multiplexed analysis. The droplet reader and the QuantaSoft Software (version 1.7, Regulatory Edition, https://www.bio-rad.com/de-de/life-science/digital-pcr/qx200-droplet-digital-pcr-system/quantasoft-software-regulatory-edition, assessed on 19 December 2023) counted the PCR-positive and PCR-negative droplets. The fraction of positive droplets was then fitted to a Poisson distribution to determine the absolute initial copy number of the target DNA molecule in the input reaction mixture in units of copies/µL.

### 2.4. Spleen and Liver Tissues from Non-Auckland Island Pigs

Frozen samples of spleen and liver tissues from dead-born (day 0), 9–30-day-old piglets and piglets older than 12 weeks from the CiMM, Munich, Germany, were used. DNA was isolated as described above and the PERV copy number was measured in both organs. 

### 2.5. Tests for PERV-C

DNA from the Auckland Island pigs was used to analyze the presence of PERV-C using a conventional PCR. Primers and conditions used were described previously as PCR1 [22,23] (Table 1). In addition, a real-time PCR was established using specific primers and probe (Table 1) [23]. 100 ng DNA and the SensiFAST Probe No-ROX kit (Meridian Bioscience Cincinnati, Newtown, OH, USA) in a 20 µL reaction volume. The cycling conditions used were initial denaturation for 5 min at 95 °C, followed by 45 amplification cycles of 95 °C for 15 s, annealing at 58 °C for 30 s, and extension at 72 °C for 30 s in a qTOWER3 G qPCR cycler (Analytik Jena, Jena, Germany). A standard curve was produced using the 510 bp amplicon of previously described PCR6 [23] (Table 1) as template.

**Table 1:** Primers and probes used for the estimation of the PERV copy number and the detection of PERV-C.

Name	Sequence	Location (Nucleotid Number)	Accession Number	Reference
PERV pol1-forward	CGACTGCCCCAAGGGTTCAA	3568–3587	HM159246	Yang et al., 2015 [24]
PERV pol2-reverse	TCTCTCCTGCAAATCTGGGCC	3803–3783		
PERV pol probe	/56FAM/CACGTACTGGAGGAGGGTCACCTG	3678–3655		
Pig actin forward	TAACCGATCCTTTCAAGCATTT			Krüger et al., 2020 [20]
Pig actin reverse	TGGTTTCAAAGCTTGCATCATA			
Pig actin probe	/5HEX/CGTGGGGATGCTTCCTGAGAAAG			
Pig GAPDH forward Pig GAPDH reverse	CCGCGATCTAATGTTCTCTTTC TTCACTCCGACCTTCACCAT	3951–3970 4022–4001	NC_010447.5 (396823)	Krüger et al., 2020 [20]
Pig GAPDH probe	/5HEX/CAGCCGCGTCCCTGAGACAC	3991–3972		
PCR1 * PERV-C forward PERV-C reverse	CTGACCTGGATTAGAACTGG ATGTTAGAGGATGGTCCTGG	6606–6625 6867–6886	AM229312	Takeuchi et al. [22], Kaulitz et al. [23]
PCR6 * PERV-C 2 forward PERV-C 2 reverse	CCAGGACCACCAAATAATGG AAGTTTTGCCCCCATTTTAGT	6435–6454 6924–6944		Kaulitz et al. [23]
Real-time PCR * PERV-C 3 forward PERV-C 3 reverse PERV-C probe	CCCCAACCCAAGGACCAG AAGTTTTGCCCCCATTTTAGT FAM-CTCTAACATAACTTCTGGATCAGACCC- BHQ1	6853–6870 6924–6944 6878–6904		

* Designation as in Kaulitz et al. [23].

This PCR was performed as follows: 100 ng of DNA template, PCR buffer I containing MgCl_2_, 0.2 mM dNTPs, and 1 unit of AmpliTaq DNA polymerase (Applied Biosystems, Inc., Waltham, MA, USA). As positive control, DNA from the PERV-C positive pig #6249) was used. Each sample was subjected to an initial denaturation of 10 min at 95 °C, followed by 45 amplification cycles (95 °C for 15 s, 58 °C for 30 s, and 72 °C for 40 s) and a final extension at 72 °C for 5 min The sensitivity of the real-time PCR was 10 copies (Supplemental Figure S1).

### 2.6. Tests for Additional Porcine Viruses

DNA, RNA, and blood from the Auckland Island pigs were used to analyze the presence of different DNA viruses and the RNA virus HEV. DNA was isolated as described above, and RNA was isolated using the RNeasy Mini kit (Qiagen, Hilden, Germany). Primers and conditions used for the PCR or real-time PCR assays have been described for PCMV/PRV [25], PCV2 [26], PCV3 [27], PLHV-1, PLHV-2, and PLHV-3 [28], as well as for HEV [28]. The Western blot and ELISA to detect antibodies against HEV using recombinant proteins were described as well [28].

## 3. Results

### 3.1. PERV Copy Number in the Genome of Adult Auckland Island Pigs

**Figure 1:**
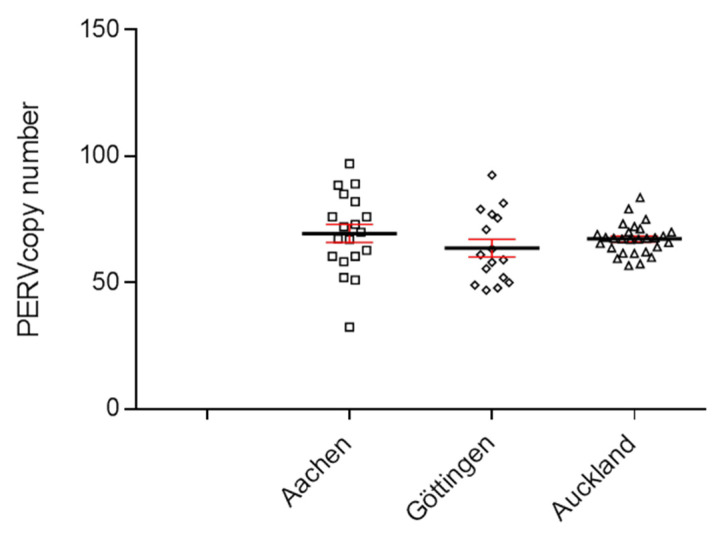
Determination of the PERVpol copy number of 30 Auckland Island pigs bred in New Zealand by ddPCR (triangels). For comparison, the PERVpol copy numbers of Göttingen minipigs (straight boxes) and Aachen minipigs (slanted boxes) are shown, which were taken from [21]. The median (black line) and the standard deviation (red line) are given. Porcine GAPDH was chosen as reference gene.

To determine the copy number of PERVs, a ddPCR was developed, which used primers binding to a highly conserved region of the polymerase gene (pol) of PERV [20,21]. DNA from 30 Auckland Island pigs, bred in New Zealand, was analyzed using the ddPCR, and a PERVpol copy number of around 65 was measured (Figure 1). This experiment was performed in parallel with the determination of the copy number in Göttingen minipigs and Aachen minipigs. The results of the copy number of these animals were already published [21], but for comparison, the results are shown here again (Figure 1). The copy number in the Auckland Island pigs was similar to the copy number of Göttingen minipigs and slightly lower compared with Aachen minipigs, investigated in parallel at the same time [21]. In this study, analyzing 16 Göttingen minipigs, a median copy number of 64 (45–93) was found, and in the case of 9 spleens and 10 livers from Aachen minipigs, a median number of 69 (34–97) was determined using GAPDH as reference gene [21].

Since the copy number of Auckland Island pigs were determined in blood cells, the copy number of Aachen minipigs in spleen and liver tissues, and the copy number of Göttingen minipigs in kidney tissue, it is difficult to compare these figures because the PERV copy number differs depending on the organ that was analyzed. This is unusual for endogenous retroviruses, which should behave like cellular genes and be constant in all tissues (see below and [20,21,29]). Furthermore, the cellular composition of the different tissues is different, and all tissues contain blood cells.

**Figure 2:**
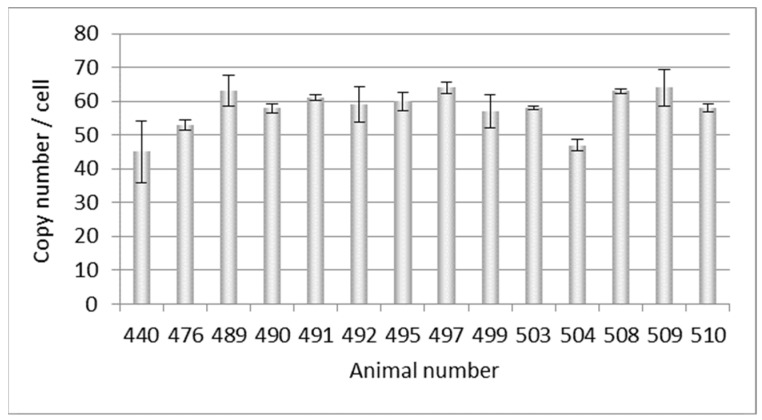
Determination of the PERVpol copy number of 14 further Auckland Island pigs bred in New Zealand by ddPCR at a later time point. The bars indicate the medium of three samples in one ddPCR reaction.

At a later point in time, DNA from another 14 Auckland Island pigs, also bred in New Zealand, was analyzed, and copy numbers between 45 and 63 were estimated (Figure 2). This experiment confirmed the previous results shown in Figure 1, but showed a broader range of copy numbers.

### 3.2. Selection of PERV-C-Negative Animals

When the 14 animals tested for the PERV copy number above (Figure 2) and another pig (494) were tested using a conventional PCR, 8 out of 15 animals (53%) were PERV-C-negative (Table 2). These investigations had the goal of selecting four PERV-C-free animals from which kidney cells were obtained, which could later be used for SCNT. PERV-C-negative cells were used for SCNT (Table 2), and consequentially, all cloned Auckland Island piglets were negative for PERV-C in the germline as tested by PCR. It is important to note that the foster mothers were positive for PERV-C and the oocytes were of unknown PERV-C status, most likely PERV-C-positive. Therefore, PERV-C was neither transmitted through recipient ooplasm during SCNT procedure nor by the embryo transfer recipient sows. This is an important finding.

When Auckland Island pigs of the F0 and F1 generation as well as of later generations were tested by conventional PCR, all animals were negative. To confirm this, a real-time PCR was established with a limit of detection of 10 copies (Supplementary Figure S1), and all tested animals were PCR-negative (no ct, not detected). 

### 3.3. PERV Copy Number in the Genome of Cell Lines from Very Young Piglets

**Figure 3:**
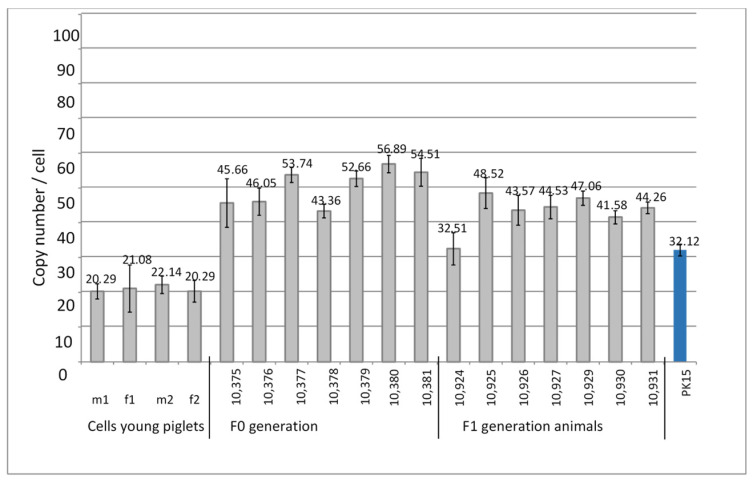
Determination of the PERVpol copy number of four kidney cell lines from very young Auckland Island pigs, and from the F0 and F1 generation of pigs obtained by somatic cell nuclear transfer (SCNT) using kidney cells from four-week-old piglets. The copy number of PK15 cells was determined as control. m, male, f, female.

DNA from four kidney cell lines from young four-week-old piglets that had been selected as PERV-C-negative was analyzed for the PERV copy number. A very low copy number of 20–22 copies was found (Table 2, Figure 3).

### 3.4. PERV Copy Number in the Genome of Auckland Island Pigs Obtained by SCNT

PERV-C-negative kidney cells from four-week-old piglets were used for SCNT into in vitro matured oocytes collected from abattoir-derived ovaries from German Landrace/Piétrain hybrid gilts. DNA from ear punches from the animals of the F0 generation at age 416 days and kidney cells of the F1 generation at age of 70 days were analyzed for the PERVpol copy number. The copy numbers of the individual pigs ranged from 32 to 57 (Figure 3), but were lower compared with the numbers of the Auckland Island pigs analyzed in the past (Figure 1 and Figure 2), and lower than other pig breeds such as the Göttingen minipigs and the Aachen minipigs [21]. However, since the copy numbers were determined in tissues of different organs, a direct comparison is not possible. PK15 cells were used as internal control in order to be able to compare the results obtained here with previous results measuring PERV copy numbers. Since different copy numbers of integrated PERV were found in different PK15 cell lines [21], here, the PK15 cells ordered from the Leibniz Institute DSMZ German Collection of Microorganisms and Cell lines, Braunschweig, Germany (ACC 640) were used, and the measured copy number was 32.12 ± 1.7 (Figure 3), which was in agreement with our previous findings [20,21]. Our investigation of different PK15 cell lines from different institutes showed cells lines with a nearly diploid chromosomal set (36–36 chromosomes) and a PERV copy number of 30 when actin was used as reference gene and 43–47 when GAPDH was used as reference gene [21]. In another PK15 cell line, a triploid chromosomal set (58–62 chromosomes) and a PERV copy number of 50 when actin was used as reference gene and 66–72 copies when GAPDH was used as reference gene were found [21]. Yang et al. [24] used PK15 cells to inactivate all PERV copies in the genome using CRISPR/Cas, and determined 62 copies in their cells.

### 3.5. Increase in the PERV Copy Number with Age in Non-Auckland Island Pigs

**Figure 4:**
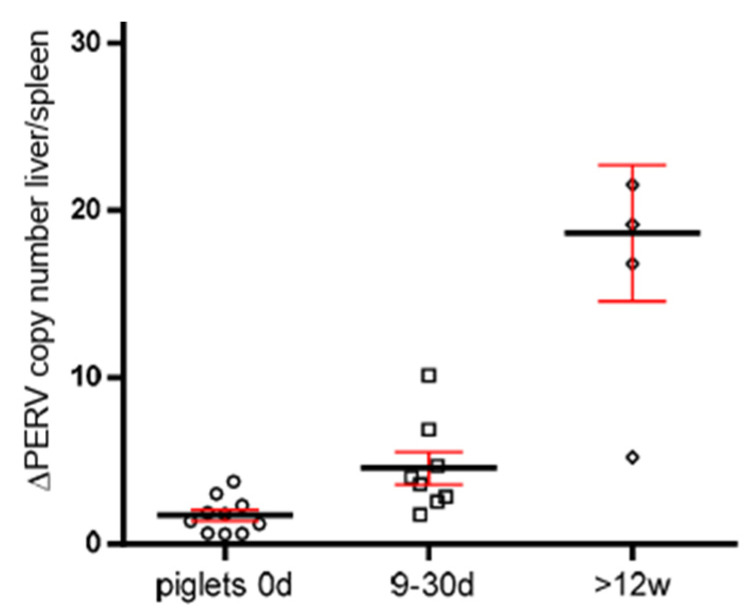
Presentation of the differences of the PERV copy number in spleen and liver of non-Auckland Island pigs depending on the age of the animals. The PERV copy number was determined in both organs of dead-born piglets (day 0) (circles) and 9–30-day-old (boxes)and 12-week-old animals (small circles) using ddPCR, and the difference was indicated depending on the age. The median (black line) and the standard deviation (red line) are given.

In order to obtain additional evidence for the increase in the PERV copy number with age, the copy numbers were determined in two different organs of dead-born (day 0), 9–30 day-old piglets and piglets older than 12 weeks from non-Auckland Island pigs (German landrace pigs), obtained from the CiMM, Munich. The copy number was determined in the spleen and liver, and the difference between the copy number was depicted depending on the age of the animals (Figure 4). This way of presentation best illustrates the result: differences in the copy number between spleen and liver were well described previously [21], and here, it was shown that they increase with age. This indicates that in addition to the copy number In the germ line, de novo integrations took place in one or the other organ. The de novo integrations were not tissue-specific: in some animals, the higher copy number was in the spleen; in others, in the liver. The difference was larger in older piglets, indicating that the copy number increased depending on the age of the animals (Figure 4).

### 3.6. Further Virological Characterization of the Auckland Island Pigs

In order to characterize the Auckland Island pigs further, PCR-based methods for the detection of PERV-C, as well as the DNA genome of PCMV/PRV, PCV1, PCV2, PCV3, PLHV-1, PLHV-2, and PLHV-3 were performed (Table 2). In addition, a reverse transcriptase (RT) PCR for the RNA genome of HEV was performed (Table 2). The tests for all these viruses were negative, with the exception of animal 476. In this animal, PLHV-3 was found by PCR. Negative were a Western blot assay and an ELISA screening for antibodies against HEV, confirming that these animals are free of HEV, as shown also by PCR. In addition to the Auckland Island pigs, the four kidney cell lines from young piglets that were four weeks old were tested for PCMV/PRV and PCV3, in addition to PERV-C, and were found negative (Table 2).

## 4. Discussion

For the first time, a determination of the number of PERV copies in Auckland Island pigs was performed using the ddPCR method. The Auckland Island pigs had been used in the first xenotransplantation trials in New Zealand and Argentina [7,8]; they are virologically well characterized [4,5,6] and are the “cleanest” pigs compared with other pig breeds. Theoretically endogenous retroviruses behave like cellular genes; they are fixed and should be identical in all tissues and cells. However, since PERVs are still active [29] and de novo integrations in somatic cells of different organs were observed, leading to different copy numbers in different organs (Figure 4), it remains unclear whether all copies are already integrated. 

The animals were free of eight porcine viruses; only in one animal was PLHV-3 detected. PLHV-3 does not harm pigs [30]. To note, the closely related PLHV-1 and -2 were not transmitted in preclinical trials transplanting pig hearts into baboons, despite the viruses being detected in the donor pigs [9]. 

In the past, it had been shown that the PERV copy number differs depending on the pig strain, on the individual animal tested, on the tissue tested, and on the method used (for review see [29]). Auckland Island pigs have been reported previously to contain 3 to 37 copies using a real-time PCR assay and a light cycler [5], and 4 to 40 copies were found using a PCR-based limited dilution assay (PLDA) [7]. In another study, relative gene dosages of PERV in Auckland Island pigs ranged from 2.8 ± 0.1 to 68 ± 1.6 at an average of 17.6 ± 10.4 using real-time PCR [31]. This indicates a generally lower copy number compared to the numbers found here, but some animals had an even higher copy number, up to 68 copies. This was higher than the copy number found in this study. In our study, we found that the PERV copy number is much lower in cells from very young piglets used for SCNT (20–22 copies) compared with the copy number in adult animals (ranging from 32 to 59 copies in the animals obtained from these cells by SCNT) (Figure 3). Unfortunately, in most studies, the age of the animals was unknown.

**Figure 5:**
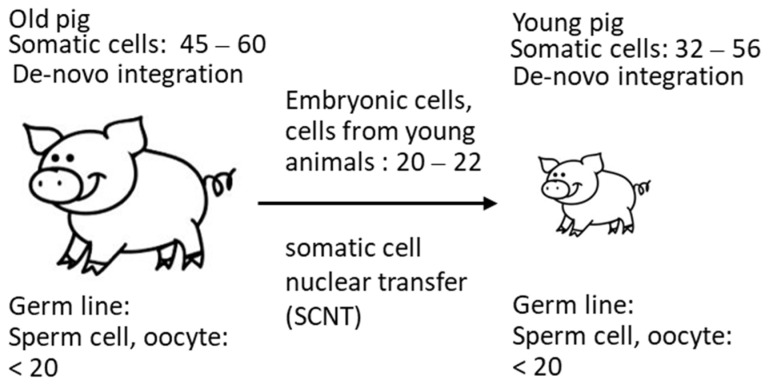
Copy number of PERV in somatic cells and in cells used for SCNT, indicating increase in the copy number in adult animals.

These data confirm that the copy number depends on the method used, as well as probably on the age of the animals. Due to de novo integrations, the number of integrated PERV proviruses differs from organ to organ (for review of numerous publications, see [24]) and even in different parts of a single organ [20] in living pigs. It is possible that not all detected copies are integrated. The finding that the copy number is lower in cells from very young animals correlates with findings in another study: when analyzing 14–21-day-old neonatal and 1–2-year-old adult Belgian Landrace pigs, a significant increase in the copy number over time was described [32]. In these animals, a difference between 5 copies and 80 copies was reported, indicating that the viruses are active and replicating in the neonatal pig. In Figure 5, a schematic presentation of these events is given. Indicated are the copy numbers found In the somatic cells of the animals and in the cells from young animals used for SCNT measured by ddPCR. Considering that copy numbers in adult animals are the result of de novo integrations in somatic cells, it logically follows that the number of proviruses in the germline can be, at most, 20 (Figure 5).

The fact that the number of integrated proviruses increased by age was also supported by a comparison of the copy number in two different tissues, spleen and liver, of non-Auckland Island pigs (Figure 4). Differences in the number of integrated copies means that in one or the other organ, additional copies appeared in addition to the copy number in the germ line. The fact that the difference increases with age indicates that more new copies were integrated with age.

As shown above, the differences in the copy number of PERV in Auckland Island pigs when measuring by different methods ([5,7,31] and this paper) did not only depend on the method. Based on our findings, the copy number increases due to de novo integrations, and it depends on the age of the animal, which was not always known to us, and therefore cannot be taken into account. It would be highly interesting to test the PERV copy number in organs of a single animal over time. Furthermore, the PERV copy number may also depend on the type of tissue and the number of PBMCs present in this tissue. The probability that de novo integrated copies can be found in the PBMCs is high since these cells are proliferating, a prerequisite for integration of gammaretroviruses. PBMCs are prone for the release of different PERVs and de novo integration evidenced by the presence of integration and release of infectious recombinant PERV-A/C from PBMCs [16,17,18]. PERV-A/C have never been found in the germ line of pigs [33]. Future investigations using separated cell populations or in situ hybridization experiments may answer this question. Furthermore, the treatment of pigs during ontogenesis with antiretroviral drugs may prevent amplification of PERVs.

Auckland Island pigs are descendants of Western pigs brought to the island [1,2]. This was confirmed by phylogenetic analyses of microsatellites and mitochondrial analyses showing that they are more closely related to European pigs than Chinese pigs [3]. The PERV copy number in Western pigs is higher compared with Chinese pigs. By analyzing 63 high-quality porcine whole-genome resequencing data, it was found that the PERV copy numbers in Chinese pigs were lower (32.0 ± 4.0) than in Western pigs (49.1 ± 6.5) [34].

From all methods ever used to count PERV copies in the DNA of pigs, such as Southern blotting [35,36], PCR titration [37], real-time PCR [38,39,40,41,42,43], fluorescence in situ hybridization (FISH) [44], PCR-based limited dilution assay [5], and genome-wide sequencing [45], ddPCR seems to be the most accurate method [29]. For example, using Southern blot analysis about 50 PERV copies were found in certain pig breeds [35,36], but PCR titration [37] or real-time PCR [38,39,40,41,42,43] detected from 1 to 98 copies. By using FISH, 19 copies of PERV-A and 13 copies of PERV-B were found in Westran pigs [44]. Using genome-wide sequencing, 20 gammaretrovirus copies and 4 betaretrovirus copies were detected in Duroc pigs [45], indicating that sequencing does not detect all integrated viruses. 

Although the favorable features of ddPCR in comparison with all other methods, including real-time PCR, have been discussed already once [29], the importance of the findings allows for a further listing of the advantages: First of all, reference standard curves are not required because the ddPCR technique provides absolute quantification based on the principles of sample partitioning and Poisson statistics. Overcoming the normalization and calibrator issues, ddPCR has shown increased precision and sensitivity. Furthermore, it is relatively insensitive to PCR inhibitors and directly provides the result of the analysis expressed as the number of copies of target per microliter of reaction [46].

Using ddPCR, fifty-nine copies were found when analyzing kidney fibroblasts from multitransgenic pigs expressing the human transgenes CD46, CD55, CD59, HO1, and A20 [21]. When wild boars in and around Berlin were analyzed using droplet digital PCR, 3 to 69 PERV copies were found. A lower copy number but a higher variability of this number was found in wild boars compared to domestic pigs, including minipigs [20].

All PCR-based methods used to estimate the PERV copy number have a common limitation in that they used primers mostly for the highly conserved pol gene. This allows them to detect all PERVs, i.e., PERV-A, PERV-B, and PERV-C. However, the detection of this sequence does not mean that there is an intact provirus able to produce infectious virus particles. Therefore, the estimated number of integrated proviruses measured with PCR-based methods tells us nothing concerning the safety of xenotransplantation, e.g., the risk posed by PERVs [47]. Only sequencing the pig genome could give more reliable information. However, the sequencing of repetitive, nearly identical sequences is always associated with problems, as has been seen with hidden sequences of the human endogenous retrovirus HERV-K [48,49]. In these investigations, in addition to 91 full-length HERV-K proviruses previously found in the human genome draft sequence, hundreds more proviruses were detected. Sequencing the genome of a domestic Duroc pig, 212 endogenous retrovirus sequences were detected, among them 9 PERV-A, 11 PERV-B, and 4 betaretrovirus copies [44], a lower number of PERVs compared with other methods [29]. 

One major result of the work was the elimination of PERV-C. Among the Auckland Island pigs analyzed in the first experiment, approximately 53% of the animals were PERV-C-negative (Table 2). PERV-C is an ecotropic virus, infecting only pig cells, and therefore does not pose a direct risk for xenotransplantation. However, recombinants between the human-tropic PERV-A and PERV-C have been observed, which are able to infect human cells and which are characterized by higher replication rates [14,15]. At present, PERV-A/C were found mainly in minipigs, either released from their PBMCs [17,18] or present in some organs of minipigs with melanomas [50], or even apparently healthy pigs [51]. Miniature swine may be a unique pig breed as they possess more copies of PERV-C sequence (typically five to nine copies) than many other pig herds (one to three copies [36]), suggesting that PERV-C may be more active in miniature swine. One exception was the finding of PERV-A/C in large farm animals with infectious diseases [19]. However, in all cases, PERV-A/C was only found in somatic cells and never in the germ line [52,53,54].

Due to our selection, there is no risk of PERV-A/C release from Auckland Island pigs generated by SCNT using cells from PERV-C-free Auckland Island pigs. These PERV-C-free pigs will be a valuable foundation for additional genetic modification in terms of future clinical xenotransplantation.

## 5. Conclusions

Auckland Island pigs had been used repeatedly as donors for pig islet cell transplantation into non-human primates and diabetic patients. In all preclinical and clinical trials, no porcine viruses, including PERV, had been transmitted.

Auckland Island pigs are well characterized; they are free of many potentially pathogenic viruses and have a PERV copy number comparable to other Western pig breeds. The copy number is much lower in cells from very young piglets and in animals obtained by SCNT using nuclei from Auckland Island pig cells. To enhance the virus safety, PERV-C-negative animals were selected. In addition, it was shown that eight other xenotransplantation-relevant pig viruses were absent in these animals. Auckland Island pigs have numerous advantages compared with other pig breeds, including their size. The organ size corresponds to that in humans, and it is therefore not necessary to inactivate the growth factor in order to achieve the desired organ size [55,56]. Other advantages are the health status, the absence of many known viruses, and maybe—due to the long isolation—also the absence of still unknown potentially xenozoonotic microorganisms.

## Figures and Tables

**Table 2:** Testing of Auckland Island pigs (animals 440–510) and kidney cells from 4-week-old piglets (w, female, m, male) for different viruses using PCR methods and a Western blot assay and an ELISA (HEV). PERV, median copy number taken from Figure 2.

Pig	Viruses *									
	PERV Copy Number	PERV-C	PCMV/PRV	HEV	HEV	HEV	PCV1/2	PCV3	PLHV-1/2	PLHV-3
		PCR	PCR	PCR	WB	ELISA	PCR	PCR	PCR	PCR
440	45	−	−	−	−	−	−	−	−	−
476	53	−	−	−	−	n.t.	−	−	−	+
489	63	+	−	−	−	−	−	−	−	−
490	58	+	−	−	−	n.t.	−	−	−	−
491	61	+	−	−	−	−	−	−	−	−
492	59	+	−	−	−	n.t.	−	−	−	−
494	n.t.	+	−	−	−	−	−	−	−	−
495	60	−	−	−	−	−	−	−	−	−
497	64	−	−	−	−	−	−	−	−	−
499	57	−	−	−	−	−	−	−	−	−
503	58	−	−	−	−	−	−	−	−	−
504	47	−	−	−	−	n.t.	−	−	−	−
508	63	+	−	−	−	n.t.	−	−	−	−
509	64	+	−	−	−	n.t.	−	−	−	−
510	58	+	−	−	−	n.t.	−	−	−	
f1	21	−	−	n.t.	n.a.	n.a.	n.t.	−	n.t.	n.t.
f2	20	−	−	n.t.	n.a.	n.a.	n.t.	−	n.t.	n.t.
m1	20	−	−	n.t.	n.a.	n.a.	n.t.	−	n.t.	n.t.
m2	22	−	−	n.t.	n.a.	n.a.	n.t.	−	n.t.	n.t.

* PERV, porcine endogenous retrovirus; PCMV/PRV, porcine cytomegalovirus/porcine roseolovirus; HEV, hepatitis E virus; PCV, porcine circovirus; PLHV, porcine lymphotropic herpesvirus, n.t., not tested, n.a., not applicable.

## Data Availability

All data supporting reported results are included in this manuscript.

## Supplementary Materials

The following supporting information can be downloaded at: https://www.mdpi.com/article/10.3390/microorganisms12010098/s1, Figure S1: Standard curve of the real-time PCR for the detection of PERV-C.

## References

[1] Gongora J., Garkavenko O., Moran C. Origins of Kune Kune and Auckland Island pigs in New Zealand. Proceedings of the 7th World Congress on Genetic Applied to Livestock Production.

[2] Robins J.H., Matisoo-Smith E., Ross H.A. (2003). The origins of the feral pigs on the Auckland Islands. J. R. Soc. N. Z..

[3] Fan B., Gongora J., Chen Y., Garkavenko O., Li Moran C. (2005). Population genetic variability and origin of Auckland Island feral pigs. J. R. Soc. N. Z..

[4] Garkavenko O., Muzina M., Muzina Z., Powels K., Elliott R.B., Croxson M.C. (2004). Monitoring for potentially xenozoonotic viruses in New Zealand pigs. J. Med. Virol..

[5] Garkavenko O., Wynyard S., Nathu D., Simond D., Muzina M., Muzina Z., Scobie L., Hector R.D., Croxson M.C., Tan P. (2008). Porcine endogenous retrovirus (PERV) and its transmission characteristics: A study of the New Zealand designated pathogen-free herd. Cell Transplant..

[6] Garkavenko O., Dieckhoff B., Wynyard S., Denner J., Elliott R.B., Tan P.L., Croxson M.C. (2008). Absence of transmission of potentially xenotic viruses in a prospective pig to primate islet xenotransplantation study. J. Med. Virol..

[7] Wynyard S., Nathu D., Garkavenko O., Denner J., Elliott R. (2014). Microbiological safety of the first clinical pig islet xenotransplantation trial in New Zealand. Xenotransplantation.

[8] Morozov V.A., Wynyard S., Matsumoto S., Abalovich A., Denner J., Elliott R. (2017). No PERV transmission during a clinical trial of pig islet cell transplantation. Virus Res..

[9] Denner J., Längin M., Reichart B., Krüger L., Fiebig U., Mokelke M., Radan J., Mayr T., Milusev A., Luther F. (2020). Impact of porcine cytomegalovirus on long-term orthotopic cardiac xenotransplant survival. Sci. Rep..

[10] Yamada K., Tasaki M., Sekijima M., Wilkinson R.A., Villani V., Moran S.G., Cormack T.A., Hanekamp I.M., Hawley R.J., Arn J.S. (2014). Porcine cytomegalovirus infection is associated with early rejection of kidney grafts in a pig to baboon xenotransplantation model. Transplantation.

[11] Sekijima M., Waki S., Sahara H., Tasaki M., Wilkinson R.A., Villani V., Shimatsu Y., Nakano K., Matsunari H., Nagashima H. (2014). Results of life-supporting galactosyltransferase knockout kidneys in cynomolgus monkeys using two different sources of galactosyltransferase knockout Swine. Transplantation.

[12] Griffith B.P., Goerlich C.E., Singh A.K., Rothblatt M., Lau C.L., Shah A., Lorber M., Grazioli A., Saharia K.K., Hong S.N. (2022). Genetically Modified Porcine-to-Human Cardiac Xenotransplantation. N. Engl. J. Med..

[13] Mohiuddin M.M., Singh A.K., Scobie L., Goerlich C.E., Grazioli A., Saharia K., Crossan C., Burke A., Drachenberg C., Oguz C. (2023). Graft dysfunction in compassionate use of genetically engineered pig-to-human cardiac xenotransplantation: A case report. Lancet.

[14] Denner J., Specke V., Thiesen U., Karlas A., Kurth R. (2003). Genetic alterations of the long terminal repeat of an ecotropic porcine endogenous retrovirus during passage in human cells. Virology.

[15] Harrison I., Takeuchi Y., Bartosch B., Stoye J.P. (2004). Determinants of high titer in recombinant porcine endogenous retroviruses. J. Virol..

[16] Wilson C.A., Wong S., Muller J., Davidson C.E., Rose T.M., Burd P. (1998). Type C retrovirus released from porcine primary peripheral blood mononuclear cells infects human cells. J. Virol..

[17] Krüger L., Kristiansen Y., Reuber E., Möller L., Laue M., Reimer C., Denner J. (2019). A comprehensive strategy for screening for xenotransplantation-relevant viruses in a second isolated population of Göttingen Minipigs. Viruses.

[18] Halecker S., Krabben L., Kristiansen Y., Krüger L., Möller L., Becher D., Laue M., Kaufer B., Reimer C., Denner J. (2022). Rare isolation of human-tropic recombinant porcine endogenous retroviruses PERV-A/C from Göttingen minipigs. Virol. J..

[19] Pal N., Baker R., Schalk S., Scobie L., Tucker A.W., Opriessnig T. (2011). Detection of porcine endogenous retrovirus (PERV) viremia in diseased versus healthy US pigs by qualitative and quantitative real-time RT-PCR. Transbound Emerg. Dis..

[20] Krüger L., Stillfried M., Prinz C., Schröder V., Neubert L.K., Denner J. (2020). Copy Number and Prevalence of Porcine Endogenous Retroviruses (PERVs) in German Wild Boars. Viruses.

[21] Fiebig U., Fischer K., Bähr A., Runge C., Schnieke A., Wolf E., Denner J. (2018). Porcine endogenous retroviruses: Quantification of the copy number in cell lines, pig breeds, and organs. Xenotransplantation.

[22] Takeuchi Y., Patience C., Magre S., Weiss R.A., Banerjee P.T., Le Tissier P., Stoye J.P. (1998). Host range and interference studies of three classes of pig endogenous retrovirus. J. Virol..

[23] Kaulitz D., Mihica D., Adlhoch C., Semaan M., Denner J. (2013). Improved pig donor screening including newly identified variants of porcine endogenous retrovirus-C (PERV-C). Arch. Virol..

[24] Yang L., Güell M., Niu D., George H., Lesha E., Grishin D., Aach J., Shrock E., Xu W., Poci J. (2015). Genome-wide inactivation of porcine endogenous retroviruses (PERVs). Science.

[25] Morozov V.A., Morozov A.V., Denner J. (2016). New PCR diagnostic systems for the detection and quantification of porcine cytomegalovirus (PCMV). Arch. Virol..

[26] Heinze J., Plotzki E., Denner J. (2016). Virus Safety of Xenotransplantation: Prevalence of Porcine Cicrovirus 2 (PCV2) in Pigs. Ann. Virol. Res..

[27] Prinz C., Stillfried M., Neubert L.K., Denner J. (2019). Detection of PCV3 in German wild boars. Virol. J..

[28] Morozov V.A., Morozov A.V., Rotem A., Barkai U., Bornstein S., Denner J. (2015). Extended Microbiological Characterization of Göttingen Minipigs in the Context of Xenotransplantation: Detection and Vertical Transmission of Hepatitis E Virus. PLoS ONE.

[29] Denner J. (2016). How Active Are Porcine Endogenous Retroviruses (PERVs)?. Viruses.

[30] Denner J. (2021). Porcine Lymphotropic Herpesviruses (PLHVs) and Xenotransplantation. Viruses.

[31] Wynyard S., Garkavenko O., Elliot R. (2011). Multiplex high resolution melting assay for estimation of Porcine Endogenous Retrovirus (PERV) relative gene dosage in pigs and detection of PERV infection in xenograft recipients. J. Virol. Methods.

[32] Mourad N.I., Crossan C., Cruikshank V., Scobie L., Gianello P. (2017). Characterization of porcine endogenous retrovirus expression in neonatal and adult pig pancreatic islets. Xenotransplantation.

[33] Bartosch B., Stefanidis D., Myers R., Weiss R., Patience C., Takeuchi Y. (2004). Evidence and consequence of porcine endogenous retrovirus recombination. J. Virol..

[34] Chen J.Q., Zhang M.P., Tong X.K., Li J.Q., Zhang Z., Huang F., Du H.P., Zhou M., Ai H.S., Huang L.S. (2022). Scan of the endogenous retrovirus sequences across the swine genome and survey of their copy number variation and sequence diversity among various Chinese and Western pig breeds. Zool Res..

[35] Le Tissier P., Stoye J.P., Takeuchi Y., Patience C., Weiss R.A. (1997). Two sets of human-tropic pig retrovirus. Nature.

[36] Patience C., Takeuchi Y., Weiss R.A. (1997). Infection of human cells by an endogenous retrovirus of pigs. Nat. Med..

[37] Patience C., Switzer W.M., Takeuchi Y., Griffiths D.J., Goward M.E., Heneine W., Stoye J.P., Weiss R.A. (2001). Multiple groups of novel retroviral genomes in pigs and related species. J. Virol..

[38] Liu G., Li Z., Pan M., Ge M., Wang Y., Gao Y. (2011). Genetic prevalence of porcine endogenous retrovirus in Chinese experimental miniature pigs. Transplant. Proc..

[39] Lee D., Lee J., Yoon J.K., Kim N.Y., Kim G.W., Park C., Oh Y.K., Kim Y.B. (2011). Rapid determination of PERV copy number from porcine genomic DNA by real-time polymerase chain reaction. Anim. Biotechnol..

[40] Yoon J.K., Choi J., Lee H.J., Cho Y., Gwon Y.D., Jang Y., Kim S., Choi H., Lee J.H., Kim Y.B. (2015). Distribution of Porcine Endogenous Retrovirus in Different Organs of the Hybrid of a Landrace and a Jeju Domestic Pig in Korea. Transplant. Proc..

[41] Zhang P., Yu P., Wang W., Zhang L., Li S., Bu H. (2010). An effective method for the quantitative detection of porcine endogenous retrovirus in pig tissues. In Vitro Cell. Dev. Biol. Anim..

[42] Quereda J.J., Herrero-Medrano J.M., Abellaneda J.M., García-Nicolás O., Martínez-Alarcón L., Pallarés F.J., Ramírez P., Muñoz A., Ramis G. (2012). Porcine endogenous retrovirus copy number in different pig breeds is not related to genetic diversity. Zoonoses Public Health.

[43] Mang R., Maas J., Chen X., Goudsmit J., van der Kuyl A.C. (2001). Identification of a novel type C porcine endogenous retrovirus: Evidence that copy number of endogenous retroviruses increases during host inbreeding. J. Gen. Virol..

[44] Lee J.H., Webb G.C., Allen R.D., Moran C. (2002). Characterizing and mapping porcine endogenous retrovirusesin Westran pigs. J. Virol..

[45] Groenen M.A., Archibald A.L., Uenishi H., Tuggle C.K., Takeuchi Y., Rothschild M.F., Rogel-Gaillard C., Park C., Milan D., Megens H.J. (2012). Analyses of pig genomes provide insight into porcine demography and evolution. Nature.

[46] Pinheiro L.B., Coleman V.A., Hindson C.M., Herrmann J., Hindson B.J., Bhat S., Emslie K.R. (2012). Evaluation of a droplet digital polymerase chain reaction format for DNA copy number quantification. Anal. Chem..

[47] Denner J. (2022). What does the PERV copy number tell us?. Xenotransplantation.

[48] Subramanian R.P., Wildschutte J.H., Russo C., Coffin J.M. (2011). Identification, characterization, and comparative genomic distribution of the HERV-K (HML-2) group of human endogenous retroviruses. Retrovirology.

[49] Contreras-Galindo R., Kaplan M.H., He S., Contreras-Galindo A.C., Gonzalez-Hernandez M.J., Kappes F., Dube D., Chan S.M., Robinson D., Meng F. (2013). HIV infection reveals widespread expansion of novel centromeric human endogenous retroviruses. Genome Res..

[50] Dieckhoff B., Puhlmann J., Büscher K., Hafner-Marx A., Herbach N., Bannert N., Büttner M., Wanke R., Kurth R., Denner J. (2007). Expression of porcine endogenous retroviruses (PERVs) in melanomas of Munich miniature swine (MMS) Troll. Vet. Microbiol..

[51] Bittmann I., Mihica D., Plesker R., Denner J. (2012). Expression of porcine endogenous retroviruses (PERV) in different organs of a pig. Virology.

[52] Scobie L., Taylor S., Wood J.C., Suling K.M., Quinn G., Meikle S., Patience C., Schuurman H.J., Onions D.E. (2004). Absence of replication-competent human-tropic porcine endogenous retroviruses in the germ line DNA of inbred miniature Swine. J. Virol..

[53] Wood J.C., Quinn G., Suling K.M., Oldmixon B.A., Van Tine B.A., Cina R., Arn S., Huang C.A., Scobie L., Onions D.E. (2004). Identification of exogenous forms of human-tropic porcine endogenous retrovirus in miniature Swine. J. Virol..

[54] Martin S.I., Wilkinson R., Fishman J.A. (2006). Genomic presence of recombinant porcine endogenous retrovirus in transmitting miniature swine. Virol. J..

[55] Hinrichs A., Riedel E.O., Klymiuk N., Blutke A., Kemter E., Längin M., Dahlhoff M., Keßler B., Kurome M., Zakhartchenko V. (2021). Growth hormone receptor knockout to reduce the size of donor pigs for preclinical xenotransplantation studies. Xenotransplantation.

[56] Goerlich C.E., Griffith B., Hanna P., Hong S.N., Ayares D., Singh A.K., Mohiuddin M.M. (2023). The growth of xenotransplanted hearts can be reduced with growth hormone receptor knockout pig donors. J. Thorac. Cardiovasc. Surg..

