# Supplementary figures and images for: Determination of the Copy Number of Porcine Endogenous Retroviruses (PERV) in Auckland Island Pigs Repeatedly Used for Clinical Xenotransplantation and Elimination of PERV-C
Source: Microorganisms. 2024 Jan 3;12(1):98. doi: 10.3390/microorganisms12010098 (PMC10820294; doi:10.3390/microorganisms12010098)

**Figure S1.** Standard curve of the real-time PCR for the detection of PERV-C.

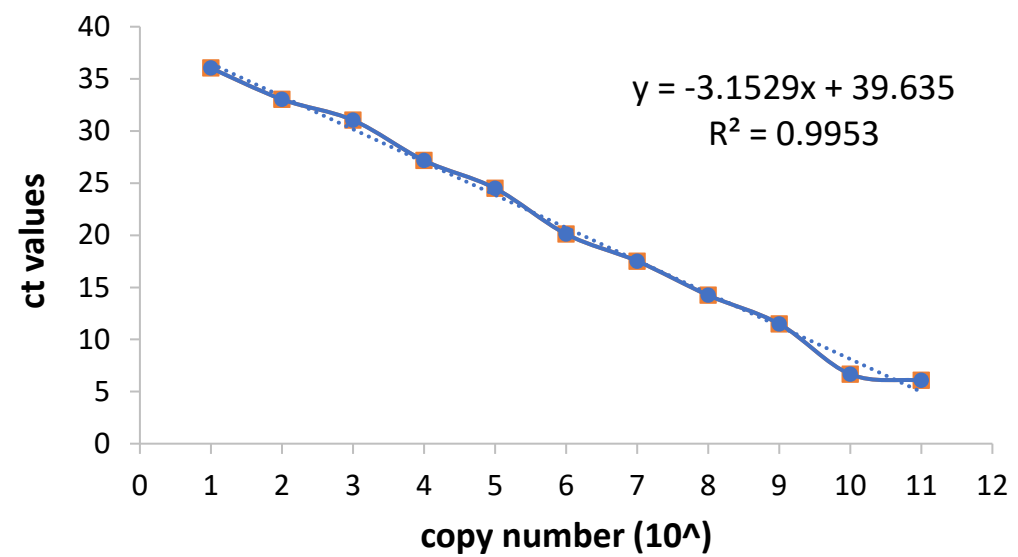

Supplement: Supplementary file 1 [file microorganisms-12-00098-s001.zip › microorganisms-2773154-supplementary.pdf]
